# The complete chloroplast genome sequence of *Isoetes baodongii* (Isoetaceae)

**DOI:** 10.1080/23802359.2024.2356128

**Published:** 2024-05-19

**Authors:** Lin Guo, Junwen Zhai, Yufeng Gu

**Affiliations:** a Ecology and Nature Conservation Institue, Chinese Academy of Forestry, Beijing, China; b Fujian Ornamental Plant Germplasm Resources Innovation & Engineering Application Research Center at College of Landscape Architecture and Art, Fujian Agriculture and Forestry University, Fuzhou, China; c Key Laboratory of National Forestry and Grassland Administration for Orchid Conservation and Utilization, the National Orchid Conservation & Research Center of Shenzhen, Shenzhen, China

**Keywords:** *Isoetes baodongii*, Isoetaceae, phylogeny, quillwort

## Abstract

*Isoetes baodongii* is a diploid species of Isoetaceae distributed in low altitude area, its megaspore ornamentation is similar to tetraploid species *I. sinensis*. We collected leaf material of *I. baodongii* and sequenced it for low depth whole genome sequence, then, a complete chloroplast genome of *I. baodongii* was assembled and annotated. This chloroplast genome has a circular structure of 145,494 bp in length with a GC content of 38.0%, comprising a large single copy (LSC) region of 91,860 bp, a pair of inverted repeat (IR) regions of 13,207 bp each, and a small single copy (SSC) region of 27,220 bp. 136 genes were annotated, including 84 protein-coding genes, 38 tRNA genes, and 8 rRNA genes. A maximum likelihood phylogeny tree was reconstructed after the sequences alignment, the result showed that *I. baodongii* formed a sister clade to the one clustered by *I. sinensis*, *I. taiwanensis* and *I. orientalis*. Although the chloroplast genome structure of *I. baodongii* is extremely similar to other species distributed in China, a well-supported phylogenetic relationship was reconstructed here, these results may provide new messages for further studies on phylogeny and evolution of vascular plant on the earth.

## Introduction

*Isoetes baodongii* Y. F. Gu, Y. H. Yan & Yi J. Lu 2021 is a diploid member of Isoetaceae, newly found in Zhejiang Province in China (Lu et al. 2021). This diploid species surprisingly grows in the wetlands with altitude less than 500 m. The height of *I. baodongii* is usually 15-40 centimeters, the megaspore ornamentation is similar to *I. sinensis* but it is a diploid species with chromosome number is 2*n* = 22 instead of tetraploid (Lu et al. 2021). Here, we assembled the complete chloroplast genome of *I. baodongii* to provide genomic resources for other researchers so that further study can be carried on this species.

**Figure 1.**
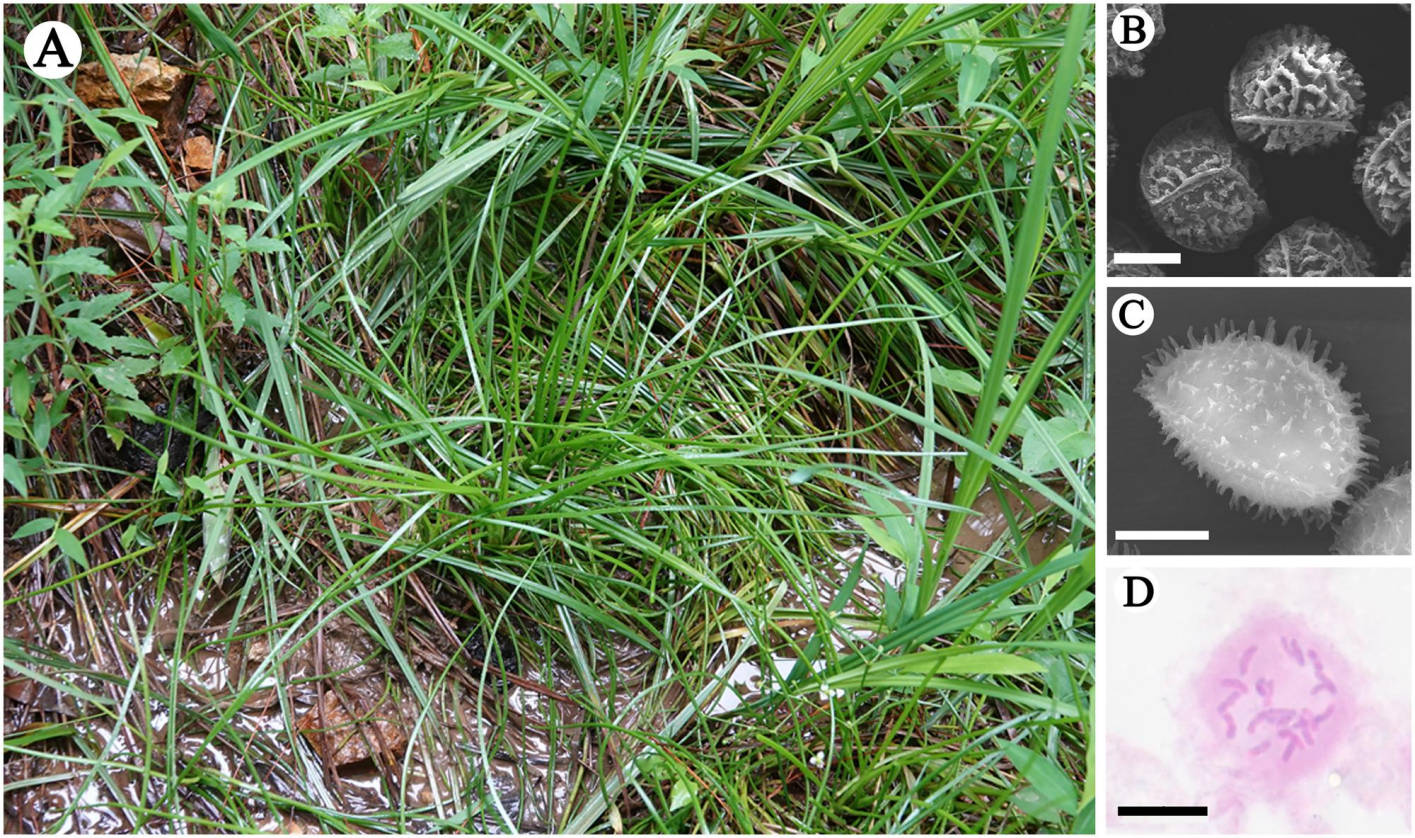
Habitat (A), megaspore (B), microspore (C) and chromosome (D) of *Isoetes baodongii*. (A) The marshes where *I. baodongii* grows are in a swampy, loamy meadow in a Danxia landform Mountain at an altitude of 230 m, all the dividual is surrounded by other plants, such as *Alternanthera philoxeroides* (Mart.) Griseb. 1879, *Cyperus rotundus* L.1753. Photoed by Yufeng Gu in Wuxie Scenic Area, Zhuji City, Zhejiang Province; (B) The ornamentation of megaspore shows as echinate-cristate, bar = 20 μm; (C) The microspore is elliptic and its surface is echinate, bar = 10 μm; (D) The chromosome number is 22, bar = 10 μm.

*Isoetes baodongii* (Figure 1) is an emergent aquatic plant growing in the marshes of Zhuji County of Zhejiang Province, China. The marshes where this diploid species grows are swampy, loamy located in a Danxia landform mountain at an altitude of 230 m. It is the third diploid Isoetaceae species reported in China and the first one found living in low altitude area. For a long time, researchers believed that diploid quillworts just distributed in high altitude area such as Qinghai-Tibet Plateau and Yunnan-Guizhou Plateau in China (Liu et al. 2004). The discovery of the diploid species is significance for the formation of tetraploid and hexaploid species in China (Lu et al. 2021; Gu et al. 2023).

## Materials and methods

Fresh leaf material was obtained from the collection sites of Wuxie Forest Farm, Zhuji County, Shaoxing City, China (longitude 120.035027 E and latitude 29.732189 N) and dried with silica. Specimens (voucher: Yufeng Gu Fern08746, shguyufeng@163.com) were deposited at the Herbarium of the Institute of Botany, Chinese Academy of Sciences (PE).

Silica-dried material was sent to Shanghai Majorbio Bio-pharm Technology Co., Ltd. (Shanghai, China) for DNA extraction and sequencing, performed on an Illumina HiSeq X platform (Illumina, San Diego, CA, USA). Briefly, genomic DNA sample was fragmented by sonication to a size of 350 bp. Then DNA fragments were endpolished, A-tailed, and ligated with the full-length adapter for Illumina sequencing, followed by further PCR amplification. After PCR products were purified by AMPure XP system (Beckman Coulter, Beverly, USA), DNA concentration was measured by Qubit 3.0 Flurometer (Invitrogen, USA), libraries were analyzed for size distribution by NGS3K/Caliper and quantified by real-time PCR (3 nM). After cluster generation, the DNA libraries were sequenced on Illumina platform and 150 bp paired-end reads were generated.

The chloroplast genome was assembled using GetOrganelle v1.7.5 (Jin et al. 2020) using default parameters, the results viewed and edited by Bandage v0.8.1 (Wick et al. 2015). The assembled chloroplast genome was annotated by Geneious Prime 2021.0.3 (https://www.geneious.com) (Kearse et al. 2012) with *I. yunguiensis* as a reference at 85% similarity.

**Figure 2.**
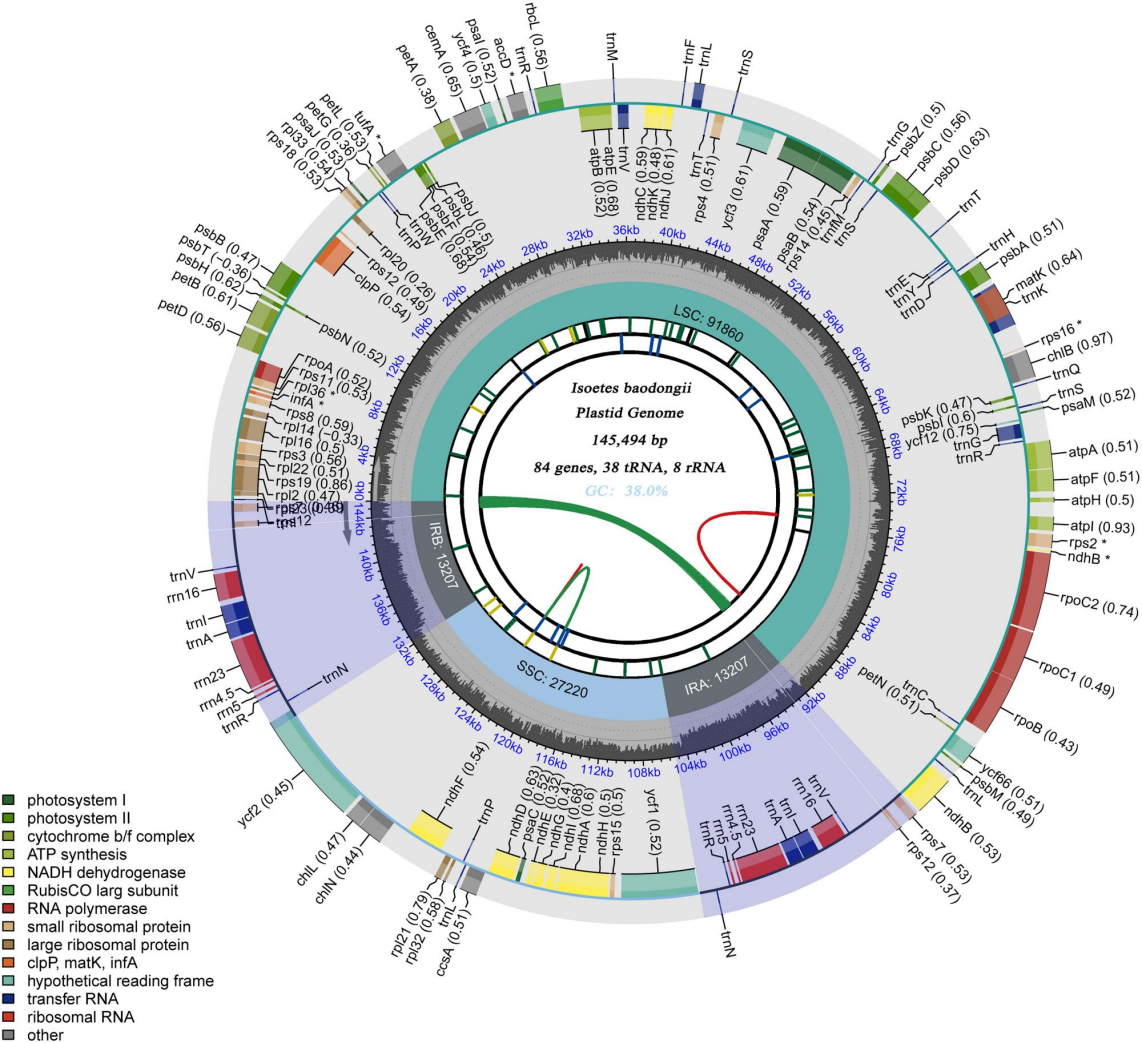
Chloroplast complete genome map of *Isoetes baodongii*. The map contains six tracks. In the first circle from the center outward, the dispersed repeats are represented. The second circle depicts the long tandem repeats. The short tandem repeats or microsatellite sequences are displayed in the third circle. The quaternary structures, namely the large single copy (LSC), small single copy (SSC), and inverted repeats (IR)a, and IRb regions are indicated in the fourth circle. The fifth circle represents the GC content. Finally, the sixth circle showcases the genes.

We drew the complete chloroplast genome map of this quillwort species (Figure 2) in OGDRAW - Draw Organelle Genome Maps (https://chlorobox.mpimp-golm.mpg.de/OGDraw.html).

To determine the phylogenetic position of *Isoetes baodongii*, 16 published complete chloroplast genomes of *Isoetes* (*I. sinensis* Palmer 1927, *I. orientalis* Hong Liu & Q. F. Wang 2005, *I. yunguiensis* Q. F. Wang & W. C. Taylor 2002, *I. hypsophila* Hand.-Mazz. 1929, *I. taiwanensis* De Vol 1972, *I. flaccida* Shuttlew. ex A.Braun 1846, *I. amazonica* A.Braun 1880, *I. butleri* Engelm. 1878, *I. melanospora* Engelm. 1877, *I. nuttallii* A.Braun ex Engelm. 1874, *I. valida* Clute 1905, *I. engelmannii* A.Braun 1846, *I. mattaponica* Musselman & W.C.Taylor 2001, *I. graniticola* D.F.Brunt. 2016, *I. malinverniana* Ces. & De Not. 1858, *I. piedmontana* (N.Pfeiff.) C.F.Reed 1965) were used to perform phylogenetic analysis; two *Huperzia* (*H. lucidula* (Ching) N. Shrestha & X. C. Zhang 2015, *H. serrata* (Thunb. ex Murray) Trevis. 1874) were set as outgroup. All these 18 sequences were downloaded from GenBank (http://www.ncbi.nlm.nih.gov/genbank/). Then, sequences were aligned using MAFFT Alignment (Katoh & Standley 2013) in Geneious Prime 2021.0.3 for phylogenetic analysis, after poorly aligned regions were excluded using Gblocks v0.91b in PhyloSuite v1.2.2 (Zhang et al. 2020); the result alignment was subjected to ML (Maximum likelihood) analyses performed using IQ-TREE v. 1.6.12 (Lam-Tung et al. 2015) with 10,000 bootstrap replicates. The best-fitting model was selected by ModelFinder (Kalyaanamoorthy et al. 2017) and implemented in IQ-TREE.

## Results

The complete chloroplast genome sequence of *Isoetes baodongii* (GenBank accession: PP213265) is 145,494 bp in length, containing a large single-copy (LSC) region of 91,860 bp, a small single-copy (SSC) region of 27,220 bp, and a pair of inverted repeats (IRs) of 13,207 bp each. A total of 136 genes were annotated, including 84 protein-coding genes, 37 transfer RNA (tRNA) genes, and 8 ribosomal RNA (rRNA) genes. The overall GC content is 38.0%.

**Figure 3.**
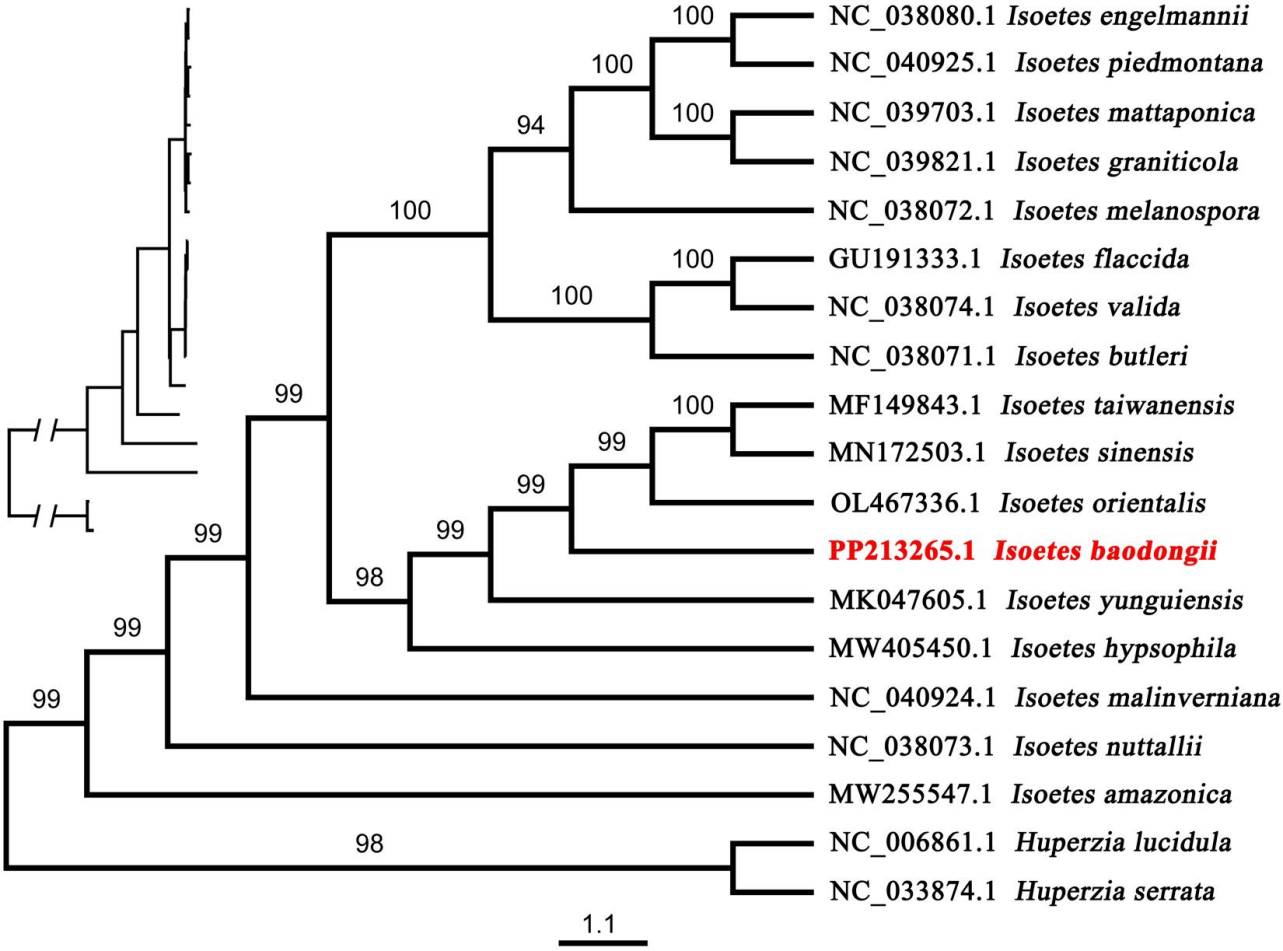
The phylogenetic position for *Isoetes baodongii* according to the maximum likelihood (ML) tree of *Isoetes* inferred from 18 chloroplast genomes by IQ-TREE. The following sequences with GenBank accession were used: *Huperzia lucidula* NC_006861.1 (Wolf et al. 2005) and *Huperzia serrata* NC_003874.1 were used as an outgroup, *Isoetes engelmannii* NC_038080.1 (Schafran et al. 2018), *Isoetes piedmontana* NC_040925.1 (Mower et al. 2019), *Isoetes mattaponica* NC_039703.1, *Isoetes graniticola* NC_039821.1, *Isoetes melanospora* NC_038072.1 (Schafran et al. 2018), *Isoetes flaccida* GU191333.1 (Karol et al. 2010), *isoetes valida* NC_038074 (Schafran et al. 2018), *Isoetes butleri* NC_038071.1 (Schafran et al. 2018), *isoetes taiwanensis* MF149843.1, *Isoetes sinensis* MN172503.1 (Xie et al. 2019), *Isoetes orientalis* OL467336.1 (Li et al. 2023), *Isoetes yunguiensis* MK047605.1 (Feng et al. 2019), *Isoetes hypsophila* MW405450.1 (Gu et al. 2021), *Isoetes malinverniana* NC_040924.1 (Mower et al. 2019), *Isoetes nuttallii* NC_038073.1 (Schafran et al. 2018), *Isoetes amazonica* MW255547.1 (Pereira et al. 2021), numbers at nodes correspond to ML bootstrap values. The scale bar represents the mean number of nucleotide acid substitutions per site. Cladogram without species names placed top left.

ML tree (Figure 3) indicated that *Isoetes amazonica*, *I. nuttallii,* and *I. malinverniana* formed separate clade solely. All the six species collected from China formed a monophyletic clade, of which *Isoetes baodongii* was a sister clade with one formed by *I. sinensis*, *I. taiwanensis* and *I. orientalis* with support ratio 99%, while *I. taiwanensis* showed a sister relationship with *I. sinensis* with 100% bootstrap support values. *I. yunguienesis* was a sister to the clade formed with the above four species *I. taiwanensis*, *I. sinensis I. orientalis* and *I. baodongii*; all the above five species formed a sister group with *I. hypsophila. Isoetes engelmannii*, *I. piedmontana*, *I. mattaponica*, *I. graniticola*, *I. melanospora*, *I. flaccida*, *I. valida* and *I. butleri* clustered as a monophyletic clade which was a sister clade with species collected from China.

## Discussion and conclusion

Five complete chloroplast genome sequences of the quillwort collected from China have been annotated (Feng et al. 2019; Xie et al. 2019; Gu et al. 2021; Lin et al. 2022; Li et al. 2023), In comparison, the chloroplast genome structure of *Isoetes baodongii* is extremely similar to other species distributed in China, such as the number of gene, CDS, tRNA. Up to now, 12 species have been published in China (Gu et al. 2023; Liu et al. 2024), we believe that the chloroplast genome of these species have been assembled and annotated but not been published yet. As the increasing use of chloroplast genomes in phylogenetic relationship analysis, more efforts on publishing chloroplast genome are still needed. When the relationship reconstructed with chloroplast genome of *Isoetes* here, we got a well-supported structure which can reveal the relationship between species. The results present here would provide additional resources for *Isoetes* further study in China even across the world. This may helpful to explore phylogeny and evolution of vascular plant on the earth.

## Supplementary Material

Supplemental Material

Supplemental Material

Supplemental Material

Supplemental Material

## Data Availability

Genome sequence data that support the findings of this study can be obtained from GenBank of NCBI (https://www.ncbi.nlm.nih.gov/) under the accession no. PP213265. Associated accession numbers listed as BioProject PRJNA1045097, SRA SRR26952483, and Bio-sample SAMN38430085.
