# Supplementary material for: The complete chloroplast genome sequence of Isoetes baodongii (Isoetaceae)
Source: Mitochondrial DNA B Resour. 2024 May 19;9(5):667–71. doi: 10.1080/23802359.2024.2356128 (PMC11107852; doi:10.1080/23802359.2024.2356128)
Supplement: Supplemental Material [file TMDN_A_2356128_SM9049.pdf]

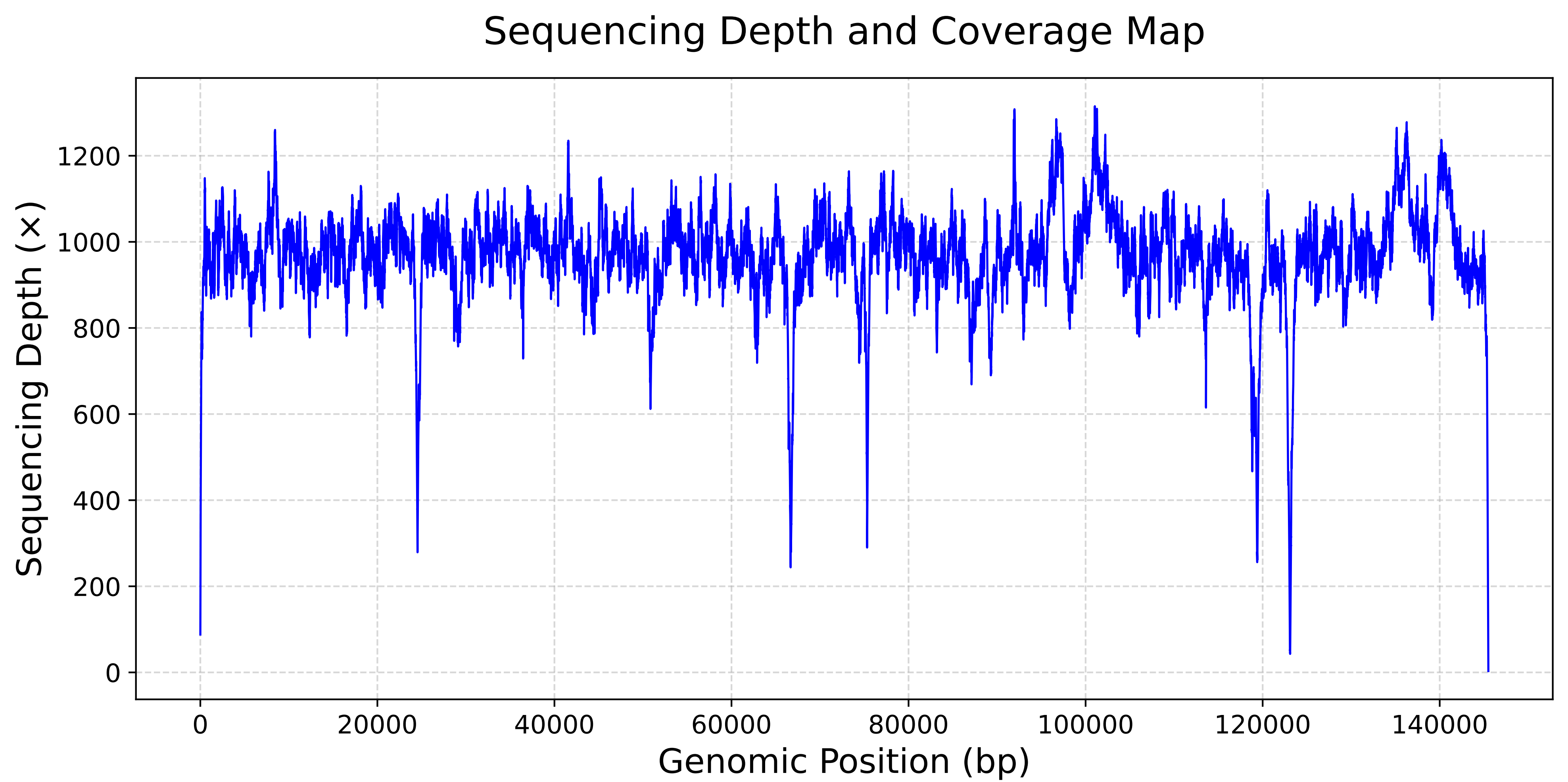

(1) Total sequence length = 145,496 bp (2) Average depth = 969.11 ×
(3) Maximum depth = 1315 × (4) Minimum depth = 3 ×
(5) Number of bases not covered by any reads: 0 bp
