# Supplementary figures and images for: The complete chloroplast genome sequence of Isoetes baodongii (Isoetaceae)
Source: Mitochondrial DNA B Resour. 2024 May 19;9(5):667–71. doi: 10.1080/23802359.2024.2356128 (PMC11107852; doi:10.1080/23802359.2024.2356128)

# Cis-splicing Genes

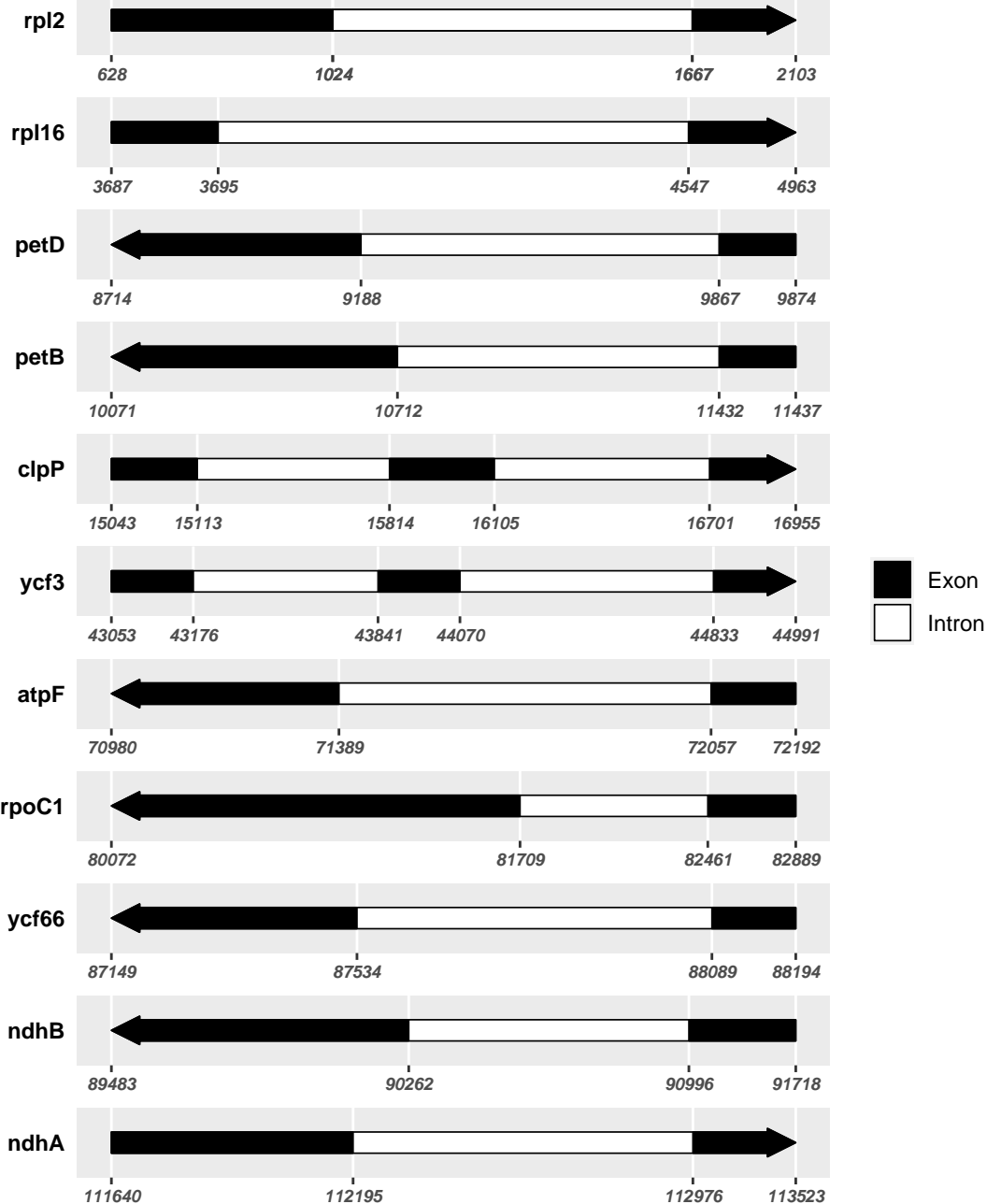

Supplement: Supplemental Material [file TMDN_A_2356128_SM9048.pdf]

# Trans-splicing Genes

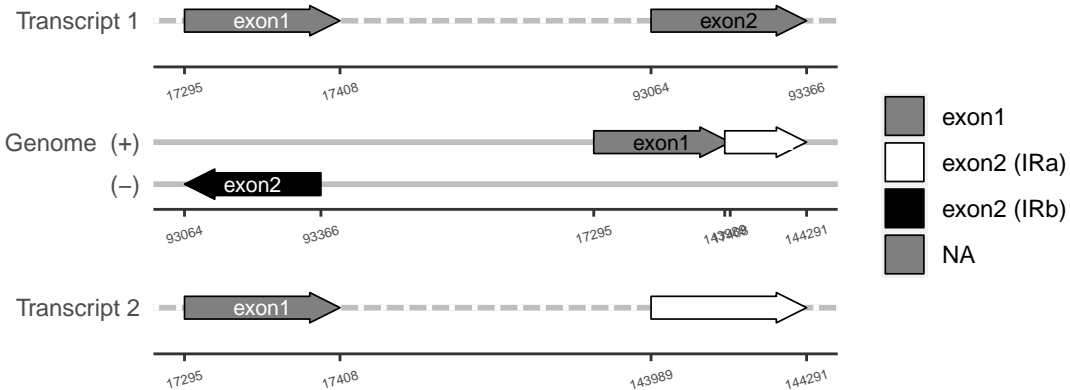

Supplement: Supplemental Material [file TMDN_A_2356128_SM9046.pdf]
