# Supplementary material for: The complete chloroplast genome sequence of Isoetes baodongii (Isoetaceae)
Source: Mitochondrial DNA B Resour. 2024 May 19;9(5):667–71. doi: 10.1080/23802359.2024.2356128 (PMC11107852; doi:10.1080/23802359.2024.2356128)
Supplement: Supplemental Material [file TMDN_A_2356128_SM9045.pdf]

美吉生物结题报告

动植物基因组 WGS 纯测序

合同编号：MJ20191105087；MJ20191211149

客户：顾钰峰

__2021__年__05__月__25__日

目 录

1 项目信息 3
2 项目流程 4
2.1 全基因重测序实验流程 4
2.2 生物信息分析流程 5
3 标准分析方法和结果 5
3.1 原始测序数据说明 5
3.2 测序碱基含量分布统计 6
3.3 测序碱基错误率分布统计 7
3.4 原始测序数据过滤 8
4 附件说明与数据下载 9
4.1 文件解压缩方法 9
4.2 文件打开或浏览方法 10
5 联系方式 10

1 项目信息

<table>
<tr><td colspan="4">项目名称</td></tr>
<tr><td colspan="4">动植物基因组 WGS 纯测序</td></tr>
<tr><td colspan="4">合同编号</td></tr>
<tr><td colspan="4">MJ20191105087；MJ20191211149</td></tr>
<tr><td colspan="4">项目样本信息</td></tr>
<tr><td>物种信息</td><td colspan="3">蕨类</td></tr>
<tr><td>基因组信息</td><td colspan="3"></td></tr>
<tr><td>备注</td><td colspan="3"></td></tr>
<tr><td colspan="4">客户信息（以上信息分析员填写，以下信息售后填写）</td></tr>
<tr><td>单位名称</td><td colspan="3">上海辰山植物园</td></tr>
<tr><td>单位地址</td><td colspan="3">上海市松江区佘山镇辰花路 3888 号科研中心 221（D4）</td></tr>
<tr><td rowspan="2">实验室负责人</td><td rowspan="2">严岳鸿</td><td>电话</td><td></td></tr>
<tr><td>邮箱</td><td></td></tr>
<tr><td rowspan="2">项目联系人</td><td rowspan="2">顾钰峰</td><td>电话</td><td>13816202969</td></tr>
<tr><td>邮箱</td><td>shguyufeng@163.com</td></tr>
<tr><td colspan="4">售后服务热线</td></tr>
<tr><td rowspan="2">结题报告审核人</td><td rowspan="2">袁波</td><td>电话</td><td>021-20725051</td></tr>
<tr><td>邮箱</td><td>rna@majorbio.com</td></tr>
<tr><td colspan="4">项目总监审批</td></tr>
<tr><td colspan="4">签名:
日期:</td></tr>
</table>

美吉生物 Majorbio
地址/Addr: 上海市浦东新区国际医学园区康新公路3399弄3号楼
网址/Web: www.majorbio.com
电话/Tel: 021-51875086
服务热线: 400-660-1216

2 项目流程

2.1 全基因重测序实验流程

样品基因组 DNA 检测合格后，利用超声波将 DNA 序列片段化形成随机片段，对片段化的 DNA 依次进行末端修复、3′端加 A、连接测序接头后，再利用磁珠吸附富集基因组长度为 400 bp 左右的片段，经过 PCR 扩增形成测序文库。建好的文库先进行文库质检，质检合格的文库用 Illumina HiSeq™ 平台进行测序，测序策略为 Illumina PE150，总测序读长为 300 bp。建库流程见图 2-1。

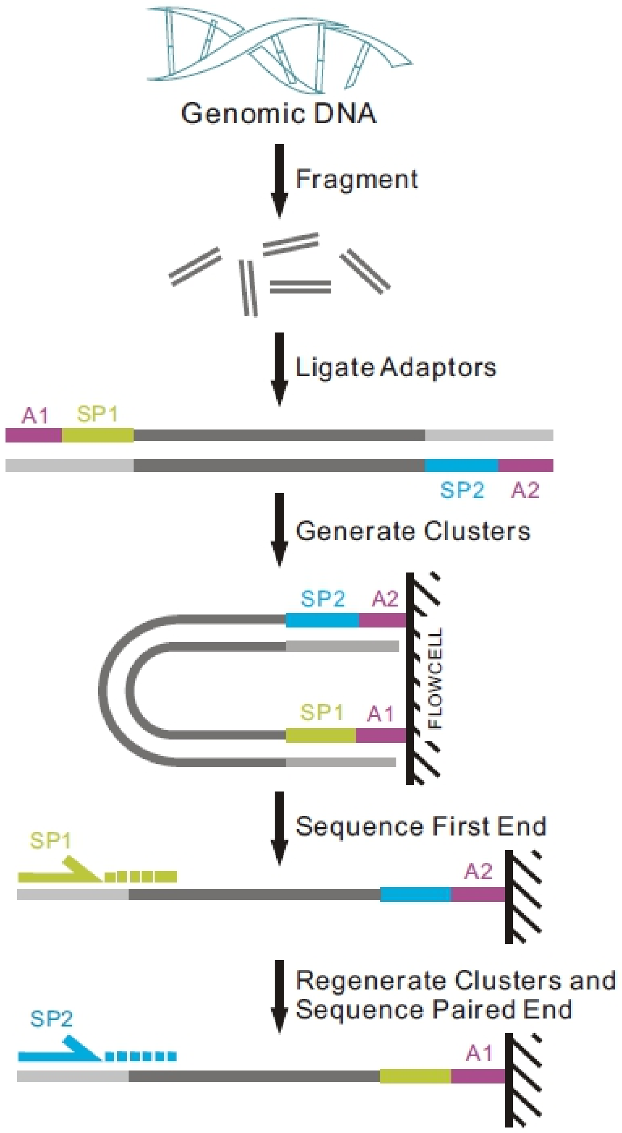

图 2-1 全基因重测序实验建库流程

2.2 生物信息分析流程

在 Illumina Hiseq™ 测序数据（Raw Data）下机之后，对下机数据进行质量控制，过滤其中低质量的数据，获得高质量的数据（Clean Data）。使用软件及版本见表 2-1。

表 2-1 生信分析软件列表

步骤	应用软件	版本
原始数据质控	Fastp	0.19.6

注：相关软件下载链接如下：

fastp：https://github.com/OpenGene/fastp

3 标准分析方法和结果

3.1 原始测序数据说明

为方便测序数据的分析、发布和共享，Illumina Hiseq™ 平台测序得到的原始图像数据经过 Base Calling 转化为序列数据，得到最原始的测序数据文件。原始数据一般存储为 FASTQ 格式。FASTQ 格式文件可记录所测读段（Reads）的碱基及其质量分数。如图 3-1 所示，FASTQ 格式以测序读段为单位进行存储，每条 Reads 在 FASTQ 格式文件中占四行，其中第一行和第三行由文件识别标志（Sequence Identifiers）和读段名（ID）组成（第一行以“@”开头而第三行以“+”开头；第三行中 ID 可以省略，但“+”不能省略），第二行为碱基序列，第四行为对应位置碱基的测序质量分数。

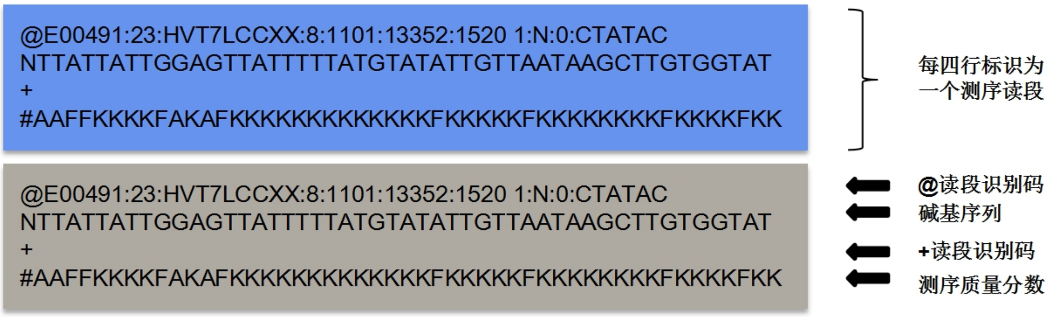

图 3-1 读段 FASTQ 数据格式示例

Illumina HiSeq™ 测序仪一个 Run 有 2 个 Flowcell，一个 Flowcell 中包含 8 个 Lane，其中一个 Lane 包含 2 列，每一列又包含 60 个 Tile，每一个 Tile 又会种下不同的 Cluster，其产生的测序文件识别标志（Sequence Identifiers）的详细说明如表 3-1 所示：

表 3-1 测序文件读段识别码说明

美吉生物 Majorbio
地址/Addr：上海市浦东新区国际医学园区康新公路3399弄3号楼 电话/Tel：021-51875086
网址/Web：www.majorbio.com 服务热线：400-660-1216

标识	英文描述
E00491	Unique instrument name
23	Run ID
HVT7LCCXX	Flowcell ID
8	Flowcell lane
1101	Tile number within the flowcell lane
13352	'x'-coordinate of the cluster within the tile
1520	'y'-coordinate of the cluster within the tile
1	Member of a pair, 1 or 2 (paired-end or mate-pair reads only)
N	Y if the read fails filter (read is bad), N otherwise
0	0 when none of the control bits are on, otherwise it is an even number
CTATAC	Index sequence

Reads 的质量分数以不同的字符来表示，在 Hiseq 平台中，将每个字符对应的 ASCII 码减去 33，即为对应的测序质量值。一般地，碱基质量从 0-40，即对应的 ASCII 码为从“!”（0+33）到“I”(40+33），碱基质量越大，可信度越高。用 e 表示测序错误率，用 Q 表示 Illumina HiSeq™ 的碱基质量值，则有下列关系：

$$Q = -10\times \lg e$$

表 3-2 测序错误率与测序质量值对应关系简明表

测序错误率（e）	测序质量值（Q）	对应 ASCII 码
5%	13	.
1%	20	5
0.1%	30	?
0.01%	40	I

Illumina 测序属于第二代测序技术，单次运行能产生数百万级的 Reads，如此海量的数据无法逐个展示每条 Reads 的质量情况；运用统计学的方法，对所有测序 Reads 的每个 Cycle 进行碱基分布和质量波动的统计，可以从宏观上直观地反映出样本的测序质量和文库构建质量。我们针对每一个样本的原始测序数据进行测序相关质量评估, 包括 A/T/G/C 碱基含量分布统计和碱基错误率分布统计。

3.2 测序碱基含量分布统计

碱基含量分布检查一般用于检测有无 AT、GC 分离现象。鉴于序列的随机性和碱基互补配对的原则，理论上每个测序循环上的 GC 含量相等、AT 含量相等，且在整个测序过程基本稳定不变，呈水平线。N 为测序仪无法判断的碱基类型。本项目中样品的碱基含量分布图如图 3-2 所示，反映出该样品的文库构建质量和测序质量均可满足后续分析。

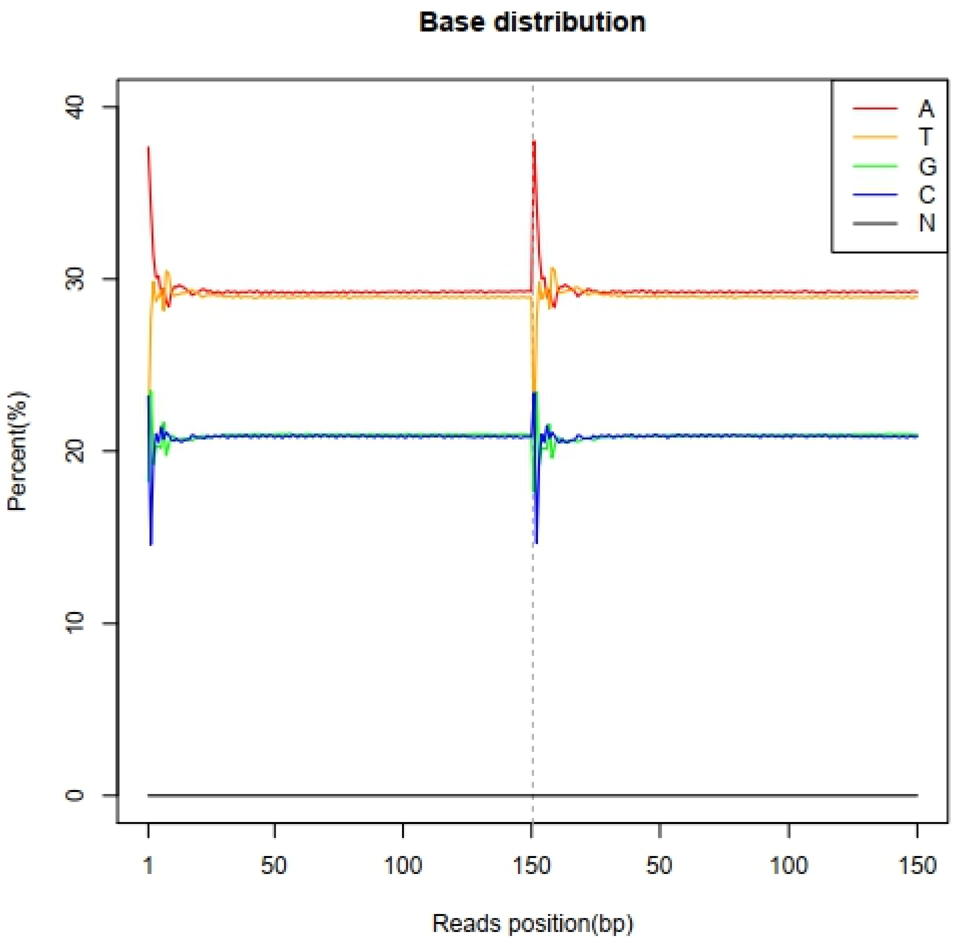

图 3-2 样品的碱基组成分布图

注： 横坐标是 Reads 碱基坐标，坐标表示 Reads 上从 5'到 3'端依次碱基的排列；纵坐标是所有 Reads 在该测序位置 A、C、G、T、N 碱基分别占的百分比，不同碱基用不同的颜色表示。序列的起始位置与测序的引物接头相连，因此 A、C、G、T 在起始端会有所波动，后面会趋于稳定。模糊碱基 N 所占比例越低，说明未知碱基数越少，测序样本受系统 AT 偏好影响越小。虚线左侧为 Read1 的统计，虚线右侧为 Read2 的统计结果。

3.3 测序碱基错误率分布统计

测序错误率会随着测序序列长度的增加而升高，这是由于测序过程中化学试剂的消耗导致的，另外，由于 Illumina Hiseq™ 测序的技术特点，测序片段前端几个 Cycles 和末端的错误率会偏高。本项目中样品的测序错误率分布如图 3-3 所示：

美吉生物 Majorbio
地址/Addr: 上海市浦东新区国际医学园区康新公路3399弄3号楼 电话/Tel: 021-51875086
网址/Web: www.majorbio.com 服务热线: 400-660-1216

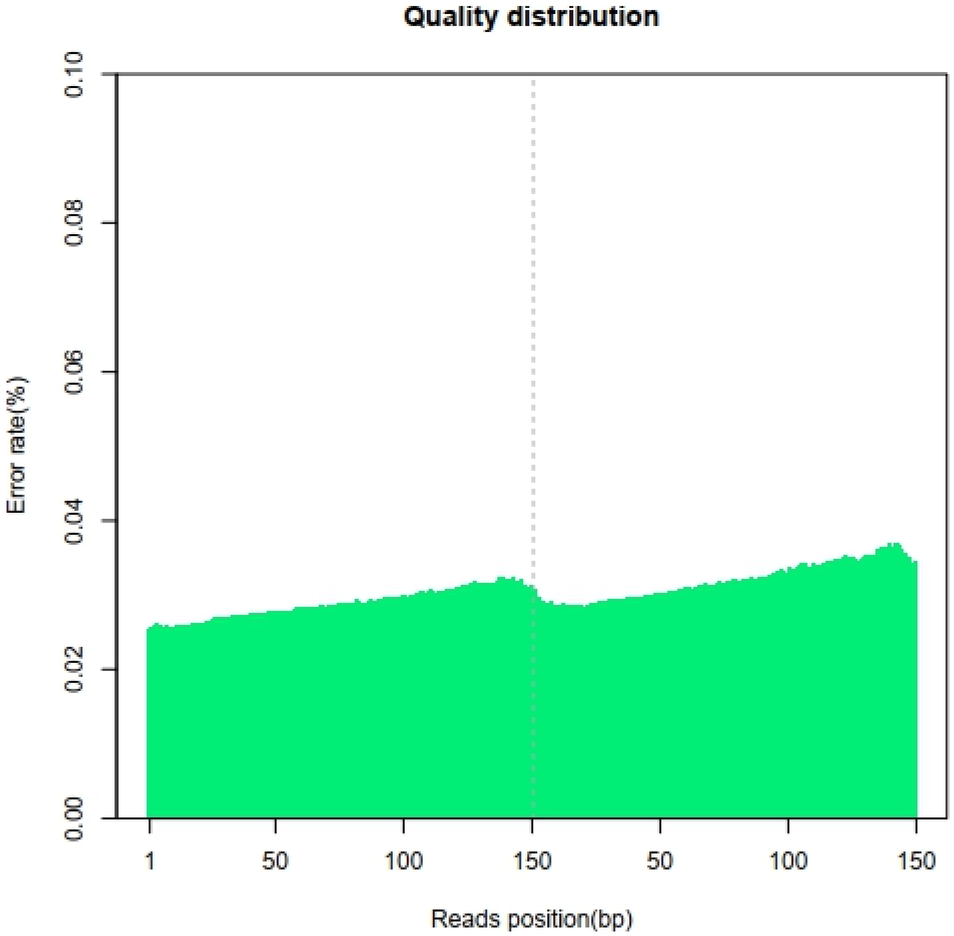

图 3-3 样品的碱基错误率分布图

注：横坐标是 Reads 碱基坐标位置，表示 Reads 上从 5'到 3'端依次碱基的排列；纵坐标是所有 Reads 在该位点处碱基的平均错误率（%）。虚线左侧为双端测序的 Read1 的错误率分布情况，虚线右侧为 Read2 的错误率分布情况。

3.4 原始测序数据过滤

利用 Illumina 的建库测序平台，构建插入片段大小为 400 bp 左右的测序文库。按照项目合同要求进行测序，由于 Illumina 的原始测序数据（Raw Data）会存在一些质量比较低的数据，所以需要进行质量过滤，获得高质量测序数据，往往过滤低质量碱基后的 Reads 长度会低于测序下机 Reads 长度，具体标准如下：

Step 1：去除 Reads 中的 Adapter 序列；

Step 2：剪切掉 5'端测序质量值低于 20 或识别为 N 的碱基；

Step 3：剪切掉 3'端测序质量值低于 3 或识别为 N 的碱基；

Step 4：以 4 个碱基为 Window，剪切掉平均质量值小于 20 的 Window 中的碱基；

Step 5：去除含 N 的比例达到 10%的 Reads；

Step 6：剪切掉超过 40%的碱基质量值低于 15 的 Reads；

Step 7：舍弃去除 Adapter 及质量修剪后长度小于 30 bp 的 Reads。

对质量剪切后的 Clean Data 别进 Reads 数、总碱基数、GC 含量和 Q30 比例的统计，详细结果见表 3-4：

表 3-4 测序质量统计表

SampleID	Clean Reads	Clean Base	Q30(%)	GC(%)
AH2405	18517248	5454088038	93.84	48.84
BDL07	15886665	4788762794	94.73	41.95
F09577	14332849	4318341970	94.31	42.55
VT123	18755918	5652615981	93.98	43.32
ZBL10995	15789505	4762102360	95.32	56.37
M3504095	33368567	10020601377	92.4	45.75
M3587572	33351641	10044825326	92.41	49.74
Fern8917	14247935	4269865416	89.91	42.03
Fern8918	15479374	4638994969	89.59	41.69
Fern9050	14548837	4360257300	90.66	44.06
Fern9068	13095023	3924476617	90.2	42.45

注：

Sample ID：样本编号；

Clean Reads：高质量的 Reads 数；

Clean Base：原始数据过滤后剩余的高质量测序数据总碱基数；

GC(%)：Clean Data 中的 GC 碱基占所有碱基的百分比；

Q30(%)：Clean Data 中质量值大于或等于 30 的碱基占所有碱基的百分比。

4 附件说明与数据下载

4.1 文件解压缩方法

所有提供的文件均为 Linux 系统下的文件，压缩包使用“tar -zcvf ”命令压缩，以下为不同系统用户解压缩的方法： Unix/Linux/Mac 用户：使用 tar -zcvf *.tar.gz 命令 ；Windows 用户：使用 WinRAR 软件解压缩

4.2 文件打开或浏览方法

如果在本附录中无特殊说明，所有提供的文件均为 Linux 系统下文本文件，Unix/Linux 用户可以使用 more 或 less 命令查看文本文件内容。对于 Windows 用户，一般文本文件可以使用写字板或者 Excel 打开。推荐使用开 PilotEdit 打开超过 10M 以上的文本文件，或选择 Unix/Linux/MacOS 系统在终端中打开相关文件

数据中可能包含部分图像文件，一般图像文件后缀名为.png、.pdf、.gif、tiff 等，对于图像文件，Windows 用户可以使用图片浏览器打开，Linux/Unix 用户使用 display 命令打开。

后缀名为 svg 的文件为文本格式描述的图像文件，Windows 用户需要安装 Adobe Illustrator 软件打开。Linux/Unix 用户可以使用 rsvg-view 命令查看。公司默认提供“pdf‘格式的矢量图，可利用"Adobe Illustrator"软件对该格式图片进行编辑。Linux 下的表格均为制表符（Tab）分割的文本，为了便于阅读，建议使用 Excel 或 OpenOffice 等办公软件用表格形式打开，打开时请用“Tab 分割”方式。

5 联系方式

	**上海总部**
**地址：**	**上海市浦东新区康新公路 3399 号时代医创园 3 号楼**
**电话：**	**021-51875086**
**E-mail：**	**dna@majorbio.com**
	**广州分公司**
**地址：**	**广州市海珠区荔福路 68 号广州市微生物所六楼西**
**电话：**	**020-61130189**
**E-mail：**	**seqgz@majorbio.com**
	**北京分公司**
**地址：**	**北京市昌平区北七家郑平路北京大数据基地 4 层 4001**
**电话：**	**010-51293026,010-51293126**
**E-mail：**	**seqbj@majorbio.com**
